# Effect of Intragenomic Sequence Heterogeneity among Multiple 16S rRNA Genes on Species Identification of *Elizabethkingia*

**DOI:** 10.1128/spectrum.01338-22

**Published:** 2022-08-29

**Authors:** Jiun-Nong Lin, Chung-Hsu Lai, Shang-Yi Lin, Ching-Chi Lee, Nan-Yao Lee, Po-Yu Liu, Chih-Hui Yang, Yi-Han Huang

**Affiliations:** a College of Medical Science and Technology, I-Shou University, Kaohsiung, Taiwan; b Department of Critical Care Medicine, E-Da Hospital, I-Shou University, Kaohsiung, Taiwan; c Division of Infectious Diseases, Department of Internal Medicine, E-Da Hospital, I-Shou University, Kaohsiung, Taiwan; d School of Medicine, College of Medicine, I-Shou University, Kaohsiung, Taiwan; e Division of Infectious Diseases, Department of Internal Medicine, Kaohsiung Medical University Hospital, Kaohsiung Medical University, Kaohsiung, Taiwan; f Clinical Medicine Research Center, National Cheng Kung University Hospital, College of Medicine, National Cheng Kung University, Tainan, Taiwan; g Division of Infectious Diseases, Department of Internal Medicine, National Cheng Kung University Hospital, Tainan, Taiwan; h School of Medicine, College of Medicine, National Cheng Kung University, Tainan, Taiwan; i Division of Infectious Diseases, Department of Internal Medicine, Taichung Veterans General Hospital, Taichung, Taiwan; j Department of Biological Science and Technology, Meiho University, Pingtung, Taiwan; Quest Diagnostics

**Keywords:** *Elizabethkingia*, 16S rRNA, phylogenetic analysis

## Abstract

Accurate identification of *Elizabethkingia* species mostly requires the use of molecular techniques, and 16S rRNA gene sequencing is generally considered the method of choice. In this study, we evaluated the effect of intraspecific diversity among the multiple copies of the 16S rRNA gene on the accuracy of species identification in the genus *Elizabethkingia.* Sequences of 16S rRNA genes obtained from the 32 complete whole-genome sequences of *Elizabethkingia* deposited in GenBank and from 218 clinical isolates collected from 5 hospitals in Taiwan were analyzed. Four or five copies of 16S rRNA were identified in the *Elizabethkingia* species with complete genome sequences. The dissimilarity among the copies of the16S rRNA gene was <1% in all *Elizabethkingia* strains. E. meningoseptica demonstrated a significantly higher rate of nucleotide variations in the 16S rRNA than did E. anophelis (*P = *0.011). Nucleotide alterations occurred more frequently in regions V2 and V6 than in other hypervariable regions (*P < *0.001). E. meningoseptica, E. anophelis, and E. argenteiflava strains were clustered distinctly in the phylogenetic tree inferred from 16S rRNA genes, and the intragenomic variation of gene sequences had no profound effect on the classification of taxa. However, E. miricola, E. bruuniana, E. ursingii, and E. occulta were grouped closely in the phylogenetic analysis, and the variation among the multiple copies of the 16S rRNA in one E. ursingii strain affected species classification. Other marker genes may be required to supplement the species classification of closely related taxa in the genus *Elizabethkingia*.

**IMPORTANCE** Incorrect identification of bacterial species would influence the epidemiology and clinical analysis of patients infected with *Elizabethkingia*. The results of the present study suggest that 16S rRNA gene sequencing should not be considered the gold standard for the accurate identification of *Elizabethkingia* species.

## INTRODUCTION

The 16S rRNA gene, a small-subunit rRNA gene, is frequently considered the gold standard for bacterial phylogenetic analysis and taxonomic classification, because it is universally present in bacteria and contains highly conservative fragments that are beneficial for designing PCR primers, hypervariable regions that enable species-level discrimination, and an adequate sequence length that can be used for sequencing (1, 2). However, bacterial genomes may contain 1 to 17 copies of the 16S rRNA gene, and sequence variations among multiple copies have been identified in many microbes (3–10). This intragenomic sequence heterogeneity may bias the identification of microbial species (9–11).

Bacteria in the genus *Elizabethkingia* are aerobic Gram-negative bacilli that can cause life-threatening infection in humans, particularly in immunocompromised patients (12, 13). Seven species comprise the genus *Elizabethkingia*, namely, E. meningoseptica, E. miricola, E. anophelis, E. bruuniana, E. ursingii, E. occulta, and E. argenteiflava (14, 15). However, species identification methods commonly employed in clinical microbiology laboratories, such as traditional biochemical techniques and matrix-assisted laser desorption ionization–time-of-flight mass spectrometry (MALDI–TOF MS), cannot correctly recognize all these species. Accurate identification can only be achieved through molecular techniques (14, 16).

Most recent studies investigating *Elizabethkingia* have performed 16S rRNA gene sequencing for species identification (13). However, differences among multiple 16S rRNA genes and the effects of intraspecific sequence variations on species identification in *Elizabethkingia* remain unclear. In this study, we investigated nucleotide variations among the multiple copies of the 16S rRNA gene in *Elizabethkingia* obtained from the National Center for Biotechnology Information (NCBI) genome sequence repository and clinical isolates collected from multiple hospitals in Taiwan. In addition, we examined the effect of intragenomic sequence heterogeneity among different 16S rRNA genes on the taxonomic classification of *Elizabethkingia* species.

## RESULTS

### Copy number and variations in 16S rRNA in whole-genome sequences.

Among the 32 whole-genome *Elizabethkingia* sequences, 6 strains (18.8%; 4 *E. meningoseptica* and 2 *E. anophelis* strains) had 4 copies of 16S rRNA genes; the other 26 *Elizabethkingia* strains (81.3%) had 5 copies of 16S rRNA genes. Intraspecific differences in sequences in 16S rRNA gene pairs for each genome are displayed in Table 1. The corresponding minimal similarity ranged from 99.41% (*E. ursingii* strain G4123) to 100% (99.94% ± 0.14% [mean ± standard deviation]). The number of nucleotide variations within a given genome ranged from 1 to 4 in most of the strains. However, *E. ursingii* strain G4123 had 15 nucleotide variations distributed in the 5 copies of the 16S rRNA gene.

**TABLE 1:** Copy number and nucleotide alterations of 16S rRNA in *Elizabethkingia* species with complete whole-genome sequences^*a*^

Species	Strain	GenBank accession no.	rRNA copy no.	rRNA difference copy no.	Nucleotide identity (%)	Nucleotide alteration(s)^*b*^
*E. meningoseptica*	KC1913	CP035809.1	4	2	99.87	G195T, A196G
*E. meningoseptica*	F2	CP050128.1	4	2	99.87	A195C, C196T
*E. meningoseptica*	G4120	CP016378.1	4	0	100	
*E. meningoseptica*	G4076	CP016376.1	4	1	99.87	G195T, A196G
*E. anophelis*	R26	CP023401.1	5	0	100	
*E. anophelis*	JM-87	CP016372.1	5	0	100	
*E. anophelis*	NUHP1	CP007547.1	5	0	100	
*E. anophelis*	JUNP 353	AP022313.1	5	0	100	
*E. anophelis*	F3201	CP016374.1	5	0	100	
*E. anophelis*	296-96	CP046080.1	5	0	100	
*E. anophelis*	SUE	CP034247.1	5	0	100	
*E. anophelis*	E6809	CP014339.1	5	0	100	
*E. anophelis*	Ag1	CP023402.1	5	0	100	
*E. anophelis*	AR4-6	CP023404.1	5	0	100	
*E. anophelis*	AR6-8	CP023403.1	5	0	100	
*E. anophelis*	FDAARGOS_198	CP023010.2	5	0	100	
*E. anophelis*	3375	CP016373.1	5	0	100	
*E. anophelis*	FDAARGOS_132	CP014020.1	5	0	100	
*E. anophelis*	FDAARGOS_134	CP014021.1	5	0	100	
*E. anophelis*	422	CP016370.1	5	1	99.74	G995T, A1008C, A1009G, A1010G
*E. anophelis*	F3543	CP014340.1	5	0	100	
*E. anophelis*	FMS-007	CP006576.1	5	0	100	
*E. anophelis*	CSID_3015183678	CP014805.2	5	0	100	
*E. anophelis*	CSID_3015183684	CP015066.2	4	0	100	
*E. anophelis*	CSID_3015183681	CP015068.2	5	0	100	
*E. anophelis*	CSID_3000521207	CP015067.2	4	0	100	
*E. miricola*	FL160902	CP040516.1	5	1	99.93	C84T
*E. miricola*	EM798-26	CP023746.1	5	0	100	
*E. miricola*	BM10	CP011059.1	5	0	100	
*E. bruuniana*	G0146	CP014337.1	5	1	99.93	C78T
*E. bruuniana*	ATCC 33958	CP035811.1	5	1	99.93	A58G
*E. ursingii*	G4123	CP016377.1	5	3	S1: 99.41 S2: 99.54 S3: 99.74	S1: A181T, A182G, A183T, C184T, C185T, A192G, A194T, A196T, A375G S2: A181G, A578G, C581G, G636T, C639T, A1120G S3: A181G, A375G, A578G, C581G

*^a^*Accessed 10 October 2021.

b Nucleotide alterations are reported in the following style: G195T indicates a G-to-T change at position 195.

### 16S rRNA in clinical isolates.

Over the study period, 218 nonduplicate isolates of *Elizabethkingia* species were collected from different patients. According to 16S rRNA gene sequencing, 15, 179, 15, 3, and 6 isolates were identified as *E. meningoseptica*, *E. anophelis*, *E. miricola*, *E. bruuniana*, and *E. ursingii*, respectively. An isolate was considered a variation if it possessed any nucleotide variation. Of the 218 clinical isolates, nucleotide variations were detected in 24 (11%) isolates, including in 5 *E. meningoseptica* isolates (33.3%), 15 *E. anophelis* isolates (8.4%), 2 *E. miricola* isolates (13.3%), and 2 *E. ursingii* isolates (33.3%). The number of nucleotide variations within a given genome ranged from 1 to 12 (Table 2). Among the 16S rRNA sequences, the least similarity between different gene copies was 99.21% (*E. anophelis* strain KMUH30), and the mean (± standard deviation) was 99.84% (±0.15%). Compared with *E. anophelis*, *E. meningoseptica* demonstrated a significantly higher rate of nucleotide variations (*P = *0.011).

**TABLE 2:** Nucleotide alterations of the 16S rRNA gene in the clinical *Elizabethkingia* isolates

Species	Strain	ANI (%)	Nucleotide alteration(s)
*E. meningoseptica*	EM653-29	99.8	C181T, G195T, A196G
*E. meningoseptica*	EM699-87	99.93	A1016C
*E. meningoseptica*	EM495-81	99.8	C181T, G195T, A196G
*E. meningoseptica*	EDC47-90	99.93	C170T
*E. meningoseptica*	VGHTC1	99.87	G195T, A196G
*E. anophelis*	EM87-63	99.93	A1256G
*E. anophelis*	EM233-27	99.93	A158G
*E. anophelis*	EM361-97	99.34	A988T, C996T, T997G, C998T, A1005G, G1006T, A1007G, C1009T, C1010T, A1022T
*E. anophelis*	EM504-35	99.93	A833G
*E. anophelis*	EM749-74	99.93	A830T
*E. anophelis*	EM960-64	99.93	A194G
*E. anophelis*	EM1049-50	99.93	C322T
*E. anophelis*	EDC49-25	99.28	A830T, A988T, C996T, T997G, C998T, A1005G, G1006T, A1007G, C1009T, C1010T, A1022T
*E. anophelis*	EDC52-15	99.93	G194T
*E. anophelis*	EDC43-35	99.93	A647G
*E. anophelis*	KMUH25	99.93	A194G
*E. anophelis*	KMUH30	99.21	A347G, A802T, A960T, C968T, G969T, C970T, A977G, G978T, A979G, C981T, C982T, A994T
*E. anophelis*	KMUH34	99.93	A805G
*E. anophelis*	KMUH38	99.93	A833G
*E. anophelis*	KMUH58	99.93	C414T
*E. miricola*	EM798-26	99.93	A1096C
*E. miricola*	KMUH27	99.93	A84G
*E. ursingii*	EM266-22	99.93	C660T
*E. ursingii*	EM514-3	99.93	C660T

### Distribution of nucleotide alterations.

**FIG 1:**
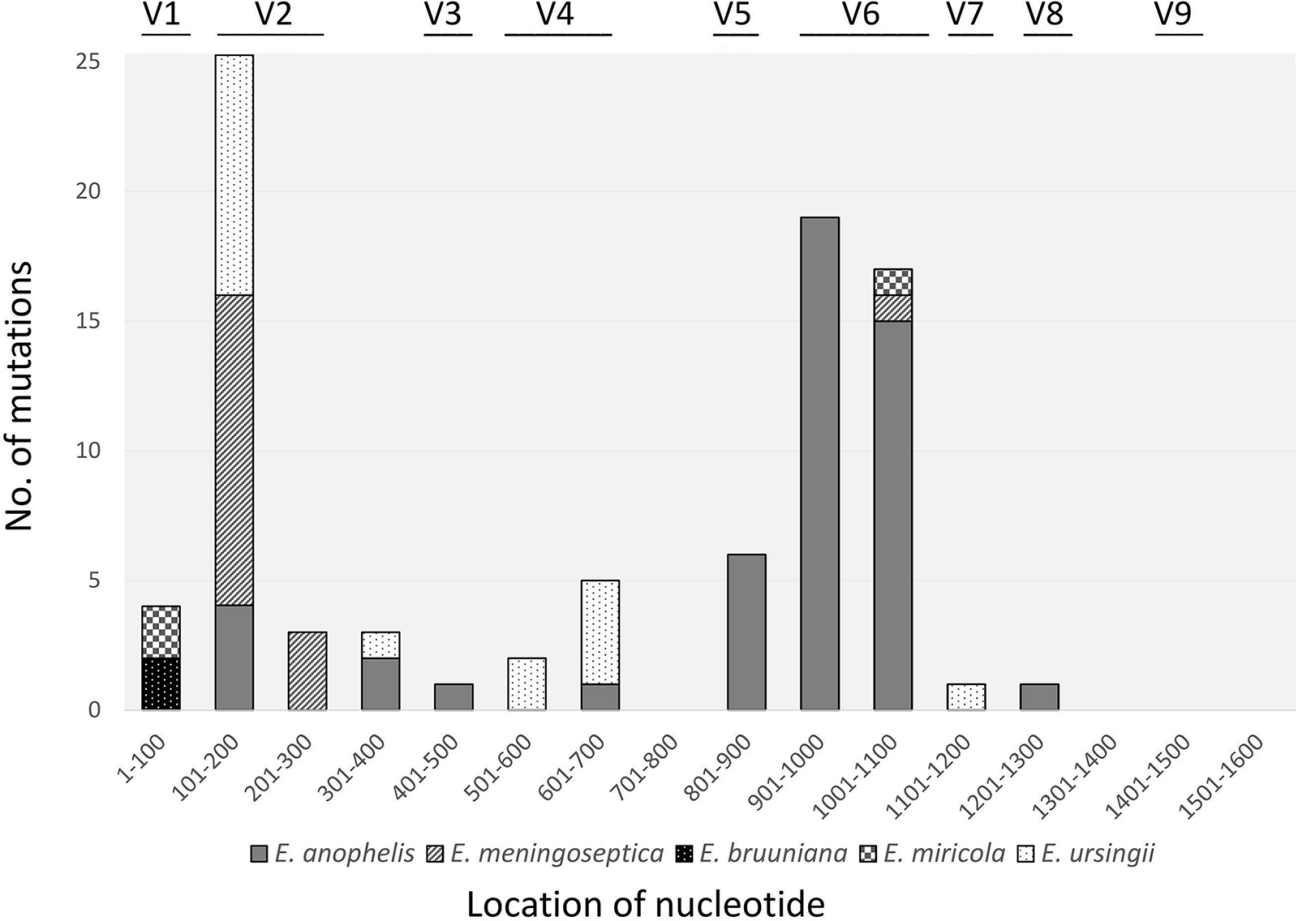
Locations and hypervariable regions of nucleotide alterations in multiple copies of the 16S rRNA genes of isolates from GenBank and clinical isolates. V1, *n* = 3; V2, *n* = 28; V3, *n* = 0; V4, *n* = 7; V5, *n* = 4; V6, *n* = 26, V7, *n* = 1; V8, *n* = 1; V9, *n* = 0.

All the alterations observed among the multiple copies of the 16S rRNA gene were single-nucleotide substitutions. Neither insertions nor deletions were identified (Table 1 and Table 2). Nucleotide variations were detected in 54 positions. Nucleotide changes were found most frequently at position 196 (*n* = 7; A↔G/T, C↔T), followed by position 195 (*n* = 6; G↔T, A↔C) and position 181 (*n* = 4; A↔G/T, C↔T). Nucleotide alterations occurred more frequently in V2 (*n* = 28, 30.8%) and V6 (*n* = 26, 28.6%) of the 16S rRNA gene than they did in other hypervariable regions (*P < *0.001) (Fig. 1).

### Phylogenetic analysis of 16S rRNA gene and species determination for strain G4123.

**FIG 2:**
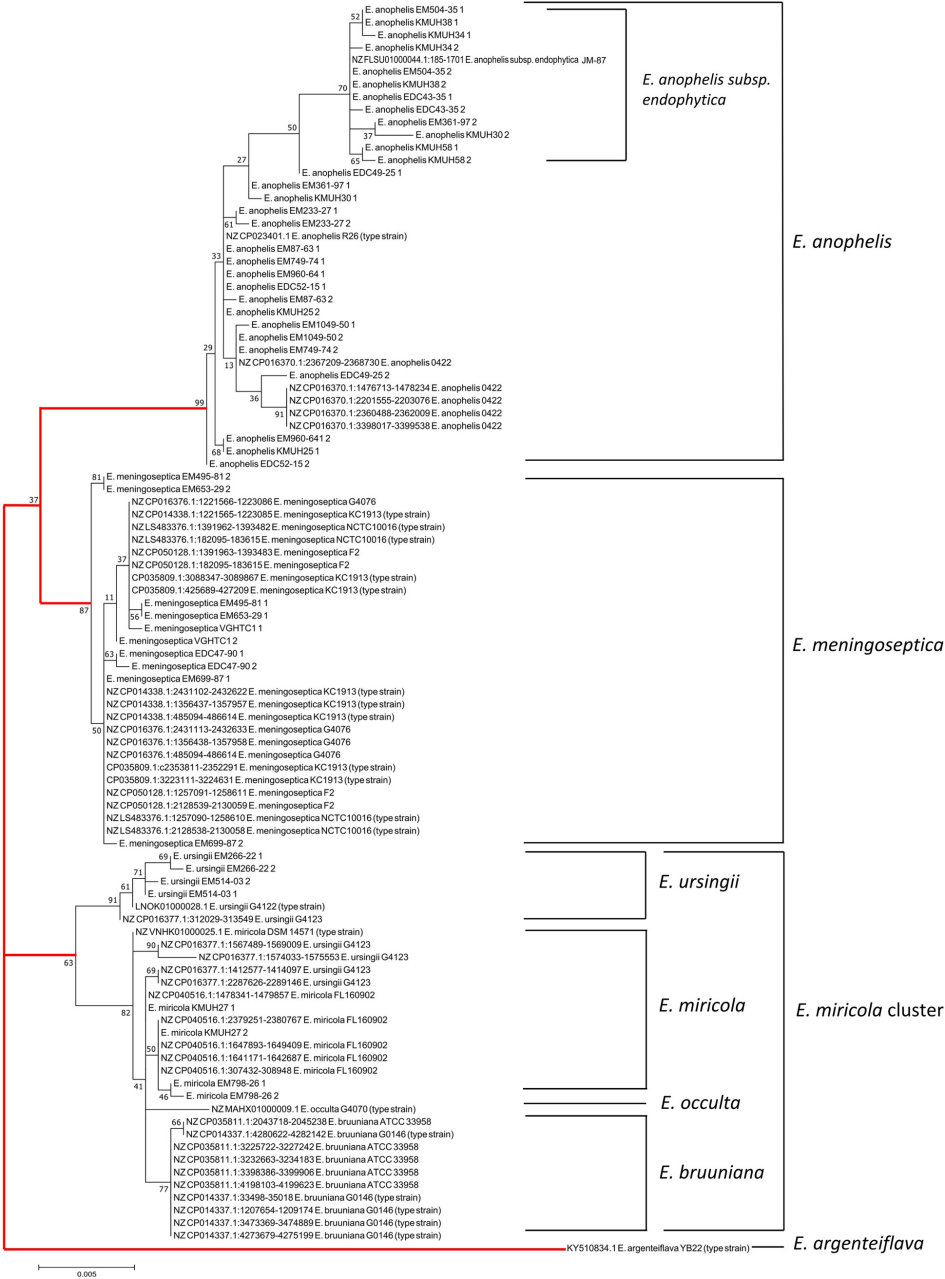
Phylogenetic analysis inferred from different copies of the 16S rRNA gene in whole-genome sequences obtained from GenBank and clinical isolates of *Elizabethkingia*. The percentages of replicate trees in which the associated taxa clustered together in a bootstrap test of 1,000 replicates are displayed next to the branches. The lines marked in red indicate the 4 major groups.

**FIG 3:**
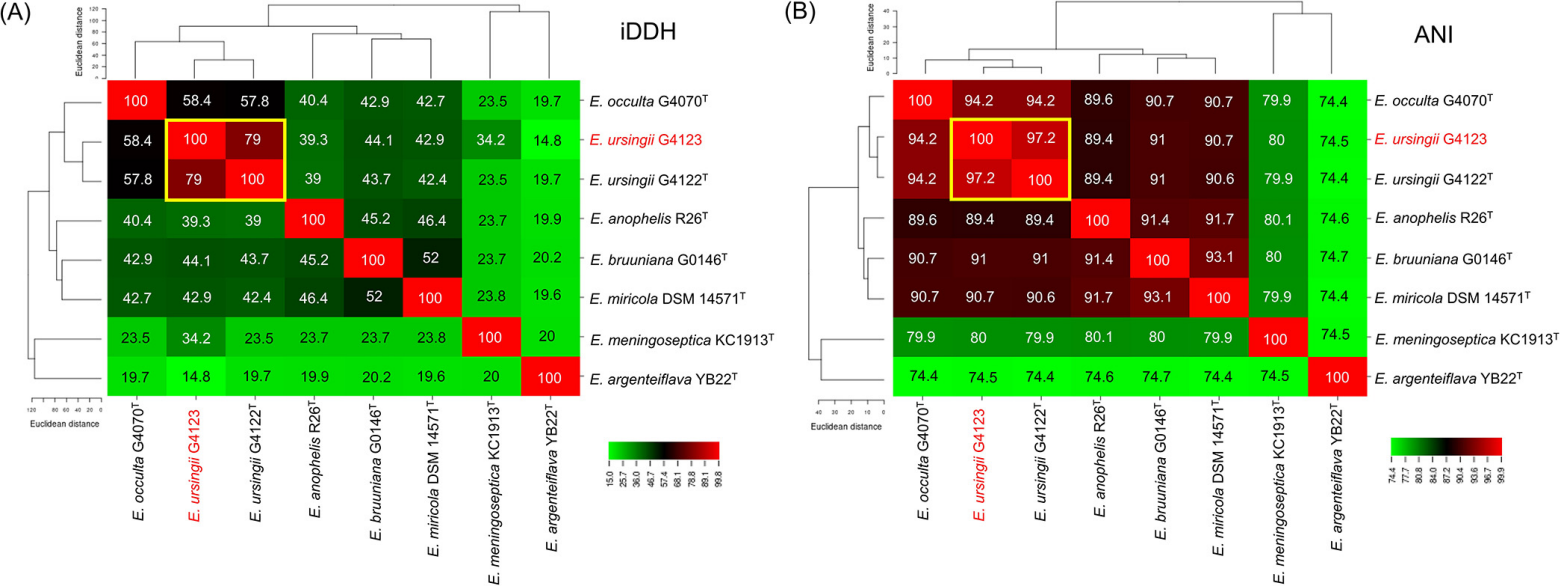
Species determination of *Elizabethkingia* strain G4123 based on whole-genome sequencing. (A) Results of *in silico* DNA-DNA hybridization (iDDH). (B) Average nucleotide identity (ANI).

A phylogenetic tree based on the 16S rRNA gene sequences for *Elizabethkingia* strains was generated to evaluate their relatedness (Fig. 2). The phylogenetic tree could be split into 4 major groups: *E. anophelis*, *E. meningoseptica*, *E. miricola* cluster, and *E. argenteiflava.* A subgroup of *E. anophelis* subsp. *endophytica* was discerned in the tree. *E. miricola*, *E. bruuniana*, *E. ursingii*, and *E. occulta* were clustered together within a close group and formed the *E. miricola* cluster. The different copies of the 16S rRNA gene did not alter the species identification in the phylogenetic tree, with the exception of *Elizabethkingia* strain G4123. Based on the whole-genome sequence analysis, *Elizabethkingia* strain G4123 was identified as *E. ursingii*, because it demonstrated 79% *in silico* DNA-DNA hybridization (iDDH) and 97.2% average nucleotide identity (ANI) with regard to *E. ursingii* type strain G4122 (Fig. 3). *E. ursingii* strain G4123 had 5 copies of the 16S rRNA gene, which were divided into 4 distinct subgroups. One copy (GenBank accession number CP016377.1, nucleotides [nt] 312029 to 313549) was close to the 16S rRNA of *E. ursingii* type strain G4122. However, the remaining 4 copies (GenBank accession number CP016377.1, nt 1412577 to 1414097, 1567489 to 1569009, 1574033 to 1575553, and 2287626 to 2289146) were close to that of *E. miricola* type strain DSM 14571 (Fig. 2).

## DISCUSSION

Species in the genus *Elizabethkingia* cannot be accurately identified using techniques based on biochemical reactions and mass spectrometry (14, 16). Therefore, sequence analysis of the bacterial 16S rRNA gene is becoming increasingly essential in clinical practice and scientific investigations, especially with respect to emerging novel microbes (17). The results of the present study demonstrated the impact of 16S rRNA gene sequence heterogeneity on species identification in the genus *Elizabethkingia*.

Our results revealed that *Elizabethkingia* strains contained 4 to 5 copies of the 16S rRNA gene, and 81.3% of strains in all species types had 5 copies. Previous studies have reported that over 80% of bacteria have more than 1 copy of the 16S rRNA gene (3–10). Some bacteria have been reported to carry more than 10 copies of the 16S rRNA gene. For example, Photobacterium profundum was reported to have 15 copies, and Paeniclostridium sordellii was reported to have 17 copies (3, 4). The number of 16S rRNA copies is believed to be related to the evolutionary response of bacteria to the physical and biological environments (8).

The 16S rRNA gene comprises highly conserved and hypervariable regions, in which numerous mutations can occur (5, 10, 18). Gene variations can be unequally distributed in diverse regions for different species (5, 10), and the hypervariable V1 to V4 regions of bacterial 16S rRNA genes have been reported to be more divergent than others (18). Regarding intragenomic heterogeneity between different copies of the 16S rRNA gene, nucleotide variations occur frequently in the V1, V2, and V6 regions (5). In the present study, 16S rRNA intraspecific heterogeneity was higher in V2 and V6 than in other regions in the genus *Elizabethkingia*. This result is compatible with the results of the above-mentioned studies (5, 18).

The sequence of the 16S rRNA gene has been widely used as an indicator for the taxonomic classification of prokaryotic microbes. The sequence variability between different copies of the 16S rRNA gene is commonly less than 1% (9, 10). Nevertheless, the intragenomic heterogeneity among different 16S rRNA gene sequences has raised concerns over the use of 16S rRNA gene sequencing for species identification. Regarding 16S rRNA gene sequencing, Pei et al. (9) analyzed 883 prokaryotic genomes of 568 bacterial species in the GenBank database and discovered that 10% of the genomes possessed >1% dissimilarity in the multiple copies of the 16S rRNA gene. Moreover, 7 species were determined to have substantial intragenomic variations in the 16S rRNA gene which led to the species being misclassified. Větrovský et al. (10) investigated 1,690 genomes in 909 bacterial species and found that 2.4% of the genomes demonstrated >1% dissimilarity between the multiple copies of the 16S rRNA gene. The highly divergent sequences of the 16S rRNA gene affect its application for taxonomic classification in some genomes (10).

In this study, the variation between the different copies of the 16S rRNA gene in all *Elizabethkingia* isolates was <1%. In the phylogenetic tree inferred from the 16S rRNA gene, *E. meningoseptica*, *E. anophelis*, and *E. argenteiflava* were clearly separately clustered. However, *E. miricola*, *E. bruuniana*, *E. ursingii*, and *E. occulta* strains were phylogenetically close, and these strains were described as the “*E. miricola* cluster” (19). Among these strains, we discovered that *Elizabethkingia* strain G4123 demonstrated considerable variations in the multiple copies of the 16S rRNA genes. The species for *Elizabethkingia* strain G4123 was confirmed to be *E. ursingii* through iDDH and ANI analysis based on whole-genome sequences. Conventional DDH has been regarded as a gold standard in prokaryote taxonomy. However, wet lab DDH is a time-consuming, labor-intensive, and potentially error-prone method (20, 21). With the advance in high-throughput sequencing technology, analysis of genomic sequences has become an accurate alternative method for conventional DDH. Among the bioinformatics methods, iDDH by *in silico* genome-to-genome comparison (20, 22) and ANI between pairwise genomes (21, 23) are considered to be accurate methods of species delineation. In the present study, we found that only 1 copy of the 16S rRNA gene approached similarity to the 16S rRNA gene of *E. ursingii* type strain G4122; the other 4 copies were closer to *E. miricola* type strain DSM 14571. Therefore, using 16S rRNA gene sequencing to discriminate between closely related strains with multiple copies of the 16S rRNA gene may have disadvantages.

Several limitations should be noted in our study. First, the whole-genome sequences of *Elizabethkingia* strains in the present study were obtained from the NCBI genome sequence repository. Although some concerns have been raised about the reliability of the public genome database (24), GenBank is considered a reliable database (25). Moreover, sequences submitted to GenBank have required review and verification for accuracy since 2012 (26). All complete whole-genome sequences of *Elizabethkingia* strains published in GenBank were submitted after 2014. Therefore, sequence data used in our study are considered accurate and reliable. Second, 16S rRNA gene sequences of clinical isolates were completed using Sanger sequencing. Therefore, we have no information on how many copies of 16S rRNA genes exist in the clinical isolates. Finally, additional genes or more whole-genome sequence studies might be needed to accurately speciate *Elizabethkingia* isolates.

In conclusion, the results of the present study indicate that the intragenomic heterogeneity among the multiple copies of 16S rRNA genes in *Elizabethkingia* species is limited. Although 16S rRNA gene sequencing can correctly identify common *Elizabethkingia* species, variations among the multiple copies of 16S rRNA genes can affect the identification of phylogenetically close species. Further studies are warranted to investigate the role of other marker genes on the taxonomic classification of these closely related taxa.

## MATERIALS AND METHODS

### Ethics.

This study was conducted in accordance with the Declaration of Helsinki and the national standards of Taiwan and was approved by the institutional review board (EMRP-109-007). The requirement for informed consent was waived because the analysis of data gathered from a public database and the retrospective analysis of clinical isolates routinely collected from patients posed no more than a minimal risk of harm to patients.

### Whole-genome sequences.

The complete whole-genome sequences of *Elizabethkingia* species were downloaded from GenBank in the NCBI genome sequence repository (https://www.ncbi.nlm.nih.gov/genome/; accessed on 10 October 2021). Thirty-four complete whole-genome sequences of *Elizabethkingia* species were available as of the time of writing, comprising 6 *E. meningoseptica* strains, 22 *E. anophelis* strains, 3 *E. miricola* strains, 2 *E. bruuniana* strains, and 1 *E. ursingii* strain. In the 6 *E. meningoseptica* strains, 3 whole-genome sequences were repeated (strain KC1913; GenBank accession number CP035809.1 is strain KC1913, GenBank accession number CP014338.1 is strain NCTC10016, and GenBank accession number LS483376.1). Finally, 32 complete genome sequences were included in the analysis.

### Strains used in this study.

Clinical isolates of *Elizabethkingia* species, obtained between 2005 and 2020, were collected from 5 hospitals in Taiwan, namely, E-Da Hospital, Kaohsiung Medical University Hospital, E-Da Cancer Hospital, National Cheng Kung University Hospital, and Taichung Veterans General Hospital. These isolates had been routinely collected from patients in accordance with clinical requirements. All isolates were initially identified as *Elizabethkingia* species by clinical microbiology laboratories using API/ID32 phenotyping kits (bioMérieux, Marcy l’Etoile, France), the Phoenix 100 ID/AST automated microbiology system (Becton, Dickinson Co., Sparks, MD, USA), the Vitek 2 automated identification system (bioMérieux), or the Vitek MALDI-TOF MS system (bioMérieux). Isolates were stored as glycerol stocks at −80°C until use.

### 16S rRNA gene sequencing of clinical isolates.

The frozen bacterial glycerol stocks were thawed and subcultured on tryptic soy agar with 5% sheep blood (Becton, Dickinson, Sparks, MD, USA) for the experiments. Bacterial DNA was prepared using a Wizard genomic DNA purification kit (Promega, Madison, WI, USA) according to the manufacturer’s instructions. All PCRs were performed using the GeneAmp 9700 system (Applied Biosystems, Foster City, CA, USA). Primers used to amplify the internal fragments of the 16S rRNA gene are listed in Table 3. PCR conditions were as described previously (27, 28). Amplicons (1,498 bp) were initially sequenced with primary sequencing primers (Table 3) by using the 3730xl DNA analyzer (Applied Biosystems). The sequencing chromatograms of the 16S rRNA genes were inspected for double peaks by using the Poly Peak Parser (http://yosttools.genetics.utah.edu/PolyPeakParser/; accessed on 12 November 2021) (29). If a region with double peaks was identified, additional sequencing using the respective supplementary primers (Table 3) was performed to verify the double peaks through chromatography.

**TABLE 3:** Primers for PCR amplification and sequencing of the 16S rRNA gene in this study

Primer purpose and name	Sequence (5′–3′)
Primers for 16S rRNA amplification	
8f	CACGGATCCAGACTTTGAT(C/T)(A/C)TGGCTCAG
1512r	GTGAAGCTTACGG(C/T)TAGCTTGTTACGACTT
Primary sequencing primers for 16S rRNA	
8f	CACGGATCCAGACTTTGAT(C/T)(A/C)TGGCTCAG
534r	ATTACCGCGGCTGCTGG
534f	CCAGCAGCCGCGGTAAT
968f	AACGCGAAGAACCTTAC
1512r	GTGAAGCTTACGG(C/T)TAGCTTGTTACGACTT
Supplementary sequencing primers for 16S rRNA	
1100f	(C/T)AACGAGCGCAACCC
1100r	GGGTTGCGCTCGTTG
337f	GACTCCTACGGGAGGC(A/T)GCAG
785f	GGATTAGATACCCTGGTA
907r	CCGTCAATTCCTTT(A/G)AGTTT
805r	GACTACCAGGGTATCTAATC
518r	GTATTACCGCGGCTGCTGG
1492r	CGGTTACCTTGTTACGACTT

### Sequence analysis and phylogenetic tree construction.

The sequences were aligned using ClustalW v2.1 with the default options in MEGA v7.0.26 (https://www.megasoftware.net/). The 16S rRNA sequences of the clinical isolates were compared with the sequence of each type strain: *E. meningoseptica* type strain KC1913 (GenBank accession number CP035809.1), *E. miricola* type strain DSM 14571 (GenBank accession number VNHK01000025.1), *E. anophelis* type strain R26 (GenBank accession number CP023401.1), *E. anophelis* subsp. *endophytica* strain JM-87 (GenBank accession number FLSU01000044.1; reclassified as *E. anophelis*), *E. bruuniana* type strain G0146 genome (GenBank accession number CP014337.1), *E. ursingii* type strain G4122 (GenBank accession number LNOK01000023.1), *E. occulta* type strain G4070 (GenBank accession number MAHX01000006.1), and *E. argenteiflava* type strain YB22 (GenBank accession number JAAABJ010000676.1). The similarity, identity, and variety between sequences were calculated using EMBOSS Water (https://www.ebi.ac.uk/Tools/psa/emboss_water/; accessed on 2 December 2021). The nucleotide identity of 16S rRNA genes between the clinical isolate and type strains was calculated. The species of *Elizabethkingia* was identified if the isolate shared the highest sequence identity and the identity was ≥99.5% (30). The locations of nucleotide alterations in the 9 hypervariable regions, namely, V1 (nt 69 to 99), 2 (nt 137 to 242), V3 (nt 433 to 497), V4 (nt 576 to 682), V5 (nt 822 to 879), V6 (nt 986 to 1043), V7 (nt 1117 to 1173), V8 (nt 1243 to 1294), and V9 (nt 1435 to 1465), of the 16S rRNA gene were mapped (31). The phylogenetic relationship was determined using MEGA v7.0.26.

### Whole-genome sequence analysis for species identification.

To accurately determine the species of *Elizabethkingia* strain G4123 (GenBank accession no. CP016377.1), iDDH and ANI values were calculated using Genome-to-Genome Distance Calculator v3.0 (20, 22) and OrthoANI v0.93 (23), respectively. An iDDH cutoff of 70% (20, 22) and an ANI cutoff of 95% (21, 23) were used as species delimitation criteria. The heat maps were produced using CIMminer (https://discover.nci.nih.gov/cimminer/, accessed on 25 June 2022).

### Statistical analysis.

We used IBM SPSS Statistics for Windows v24 (IBM Corp., Armonk, NY, USA) to perform statistical analysis with Fisher’s exact test for categorical variables and Student's *t* test for continuous variables. A two-tailed *P* value of <0.05 was considered statistically significant. The maximum-likelihood method based on the Jukes-Cantor model (JC69) was used to estimate the evolutionary distance in the phylogenetic tree constructed with MEGA v7.0.26.

### Data availability.

The GenBank accession numbers of 16S rRNA gene sequences of the clinical *Elizabethkingia* isolates in the present study are available in Table S1 in the supplemental material.
