# Supplementary material for: Effect of Intragenomic Sequence Heterogeneity among Multiple 16S rRNA Genes on Species Identification of Elizabethkingia
Source: Microbiol Spectr. 2022 Aug 29;10(5):e01338-22. doi: 10.1128/spectrum.01338-22 (PMC9604143; doi:10.1128/spectrum.01338-22)
Supplement: Supplemental file 1 — Supplemental material. Download spectrum.01338-22-s0001.pdf, PDF file, 0.1 MB [file spectrum.01338-22-s0001.pdf]

Table S1. Strains and accession numbers of 16S rRNA genes of clinical *Elizabethkingia* isolates in the present study

**Strain**	**GenBank accession number**
EM37-36	ON714654.1
EM60-32	ON714655.1
EM67-05	ON714656.1
EM72-72	ON714657.1
EM86-50	ON714658.1
EM90-61	ON714659.1
EM98-05	ON714660.1
EM99-75	ON714661.1
EM141-42	ON714662.1
EM151-25	ON714663.1
EM160-82	ON714664.1
EM165-95	ON714665.1
EM166-91	ON714666.1
EM184-18	ON714667.1
EM197-78	ON714668.1
EM199-61	ON714669.1
EM204-47	ON714670.1
EM239-83	ON714671.1
EM248-64	ON714672.1
EM261-59	ON714673.1
EM265-62	ON714674.1
EM267-19	ON714675.1
EM331-28	ON714676.1
EM356-57	ON714677.1
EM392-71	ON714678.1
EM442-68	ON714679.1
EM457-24	ON714680.1
EM458-14	ON714681.1
EM462-61	ON714682.1
EM521-29	ON714683.1
EM550-81	ON714684.1
EM551-63	ON714685.1
EM551-80	ON714686.1
EM554-21	ON714687.1

EM564-70	ON714688.1
EM566-01	ON714689.1
EM579-56	ON714690.1
EM579-68	ON714691.1
EM603-41	ON714692.1
EM642-82	ON714693.1
EM647-28	ON714694.1
EM647-31	ON714695.1
EM649-87	ON714696.1
EM657-50	ON714697.1
EM657-91	ON714698.1
EM666-89	ON714699.1
EM703-45	ON714700.1
EM703-77	ON714701.1
EM704-87	ON714702.1
EM711-82	ON714703.1
EM717-18	ON714704.1
EM717-26	ON714705.1
EM717-30	ON714706.1
EM730-50	ON714707.1
EM755-100	ON714708.1
EM767-89	ON714709.1
EM769-37	ON714710.1
EM770-49	ON714711.1
EM772-81	ON714712.1
EM782-03	ON714713.1
EM792-75	ON714714.1
EM800-59	ON714715.1
EM808-63	ON714716.1
EM833-85	ON714717.1
EM852-48	ON714718.1
EM853-49	ON714719.1
EM854-77	ON714720.1
EM861-61	ON714721.1
EM861-69	ON714722.1
EM863-79	ON714723.1
EM870-91	ON714724.1

EM919-93	ON714725.1
EM939-34	ON714726.1
EM943-07	ON714727.1
EM945-17	ON714728.1
EM981-34	ON714729.1
EM984-29	ON714730.1
EM986-96	ON714731.1
EM989-63	ON714732.1
EM990-96	ON714733.1
EM991-03	ON714734.1
EM993-93	ON714735.1
EM998-58	ON714736.1
EM999-65	ON714737.1
EM1006-53	ON714738.1
EM1022-35	ON714739.1
EM1023-67	ON714740.1
EM1027-67	ON714741.1
EM1037-10	ON714742.1
EM1042-67	ON714743.1
EM1045-85	ON714744.1
EM1048-16	ON714745.1
EM1050-03	ON714746.1
EM1054-01	ON714747.1
EM1059-55	ON714748.1
EM1064-93	ON714749.1
EM1065-50	ON714750.1
EM1070-90	ON714751.1
EM1071-77	ON714752.1
EDC15-70	ON714753.1
EDC23-98	ON714754.1
EDC32-52	ON714755.1
EDC41-75	ON714756.1
EDC44-32	ON714757.1
EDC45-72	ON714758.1
EDC46-69	ON714759.1
EDC53-89	ON714760.1
EDC57-50	ON714761.1

VGHTC2	ON714762.1
VGHTC3	ON714763.1
VGHTC6	ON714764.1
VGHTC9	ON714765.1
VGHTC10	ON714766.1
VGHTC11	ON714767.1
VGHTC12	ON714768.1
KMUH13	ON714769.1
KMUH14	ON714770.1
KMUH15	ON714771.1
KMUH16	ON714772.1
KMUH17	ON714773.1
KMUH18	ON714774.1
KMUH19	ON714775.1
KMUH20	ON714776.1
KMUH21	ON714777.1
KMUH22	ON714778.1
KMUH23	ON714779.1
KMUH26	ON714780.1
KMUH28	ON714781.1
KMUH35	ON714782.1
KMUH37	ON714783.1
KMUH39	ON714784.1
KMUH40	ON714785.1
KMUH41	ON714786.1
KMUH42	ON714787.1
KMUH43	ON714788.1
KMUH45	ON714789.1
KMUH46	ON714790.1
KMUH47	ON714791.1
KMUH48	ON714792.1
KMUH49	ON714793.1
KMUH50	ON714794.1
KMUH51	ON714795.1
KMUH52	ON714796.1
KMUH53	ON714797.1
KMUH54	ON714798.1

KMUH56	ON714799.1
KMUH57	ON714800.1
KMUH59	ON714801.1
KMUH60	ON714802.1
NCKU61	ON714803.1
NCKU62	ON714804.1
NCKU63	ON714805.1
NCKU64	ON714806.1
NCKU65	ON714807.1
NCKU66	ON714808.1
NCKU67	ON714809.1
NCKU68	ON714810.1
NCKU69	ON714811.1
NCKU70	ON714812.1
NCKU71	ON714813.1
NCKU72	ON714814.1
NCKU73	ON714815.1
NCKU74	ON714816.1
NCKU75	ON714817.1
EM87-63 sequence 1	ON714818.1
EM87-63 sequence 2	ON714819.1
EM233-27 sequence 1	ON714820.1
EM233-27 sequence 2	ON714821.1
EM361-97 sequence 1	ON714822.1
EM361-97 sequence 2	ON714823.1
EM504-35 sequence 1	ON714824.1
EM504-35 sequence 2	ON714825.1
EM749-74 sequence 1	ON714826.1
EM749-74 sequence 2	ON714827.1
EM960-64 sequence 1	ON714828.1
EM960-64 sequence 2	ON714829.1
EM1049-50 sequence 1	ON714830.1
EM1049-50 sequence 2	ON714831.1
EDC43-35 sequence 1	ON714832.1
EDC43-35 sequence 2	ON714833.1
EDC49-25 sequence 1	ON714834.1
EDC49-25 sequence 2	ON714835.1

EDC52-15 sequence 1	ON714836.1
EDC52-15 sequence 2	ON714837.1
KMUH25 sequence 1	ON714838.1
KMUH25 sequence 2	ON714839.1
KMUH30 sequence 1	ON714840.1
KMUH30 sequence 2	ON714841.1
KMUH34 sequence 1	ON714842.1
KMUH34 sequence 2	ON714843.1
KMUH38 sequence 1	ON714844.1
KMUH38 sequence 2	ON714845.1
KMUH58 sequence 1	ON714846.1
KMUH58 sequence 2	ON714847.1
EM168-80	ON714848.1
EM249-06	ON714849.1
EM558-41	ON714850.1
EM806-31	ON714851.1
EM806-35	ON714852.1
EM1013-89	ON714853.1
EM1073-59	ON714854.1
EDC32-42	ON714855.1
EDC54-90	ON714856.1
KMUH36	ON714857.1
EM495-81 sequence 1	ON714858.1
EM495-81 sequence 2	ON714859.1
EM653-29 sequence 1	ON714860.1
EM653-29 sequence 2	ON714861.1
EM699-87 sequence 1	ON714862.1
EM699-87 sequence 2	ON714863.1
EDC47-90 sequence 1	ON714864.1
EDC47-90 sequence 2	ON714865.1
VGHTC1 sequence 1	ON714866.1
VGHTC1 sequence 2	ON714867.1
EM20-50	ON714868.1
EM334-11	ON714869.1
EM828-05	ON714870.1
EM863-68	ON714871.1
EM891-63	ON714872.1

EM980-84	ON714873.1
EM1028-21	ON714874.1
EDC55-54	ON714875.1
VGHTC4	ON714876.1
VGHTC7	ON714877.1
VGHTC8	ON714878.1
KMUH33	ON714879.1
KMUH44	ON714880.1
EM798-26 sequence 1	ON714881.1
EM798-26 sequence 2	ON714882.1
KMUH27 sequence 1	ON714883.1
KMUH27 sequence 2	ON714884.1
EM455-89	ON714885.1
EM896-35	ON714886.1
KMUH55	ON714887.1
EM229-20	ON714888.1
KMUH29	ON714889.1
KMUH31	ON714890.1
KMUH32	ON714891.1
EM266-22 sequence 1	ON714892.1
EM266-22 sequence 2	ON714893.1
EM514-03 sequence 1	ON714894.1
EM514-03 sequence 2	ON714895.1
